# 

**Published:** 1887-08-27

**Authors:** 


The Hospital.

An Institution, Family, and Parochial Journal of Hospitals, Asylums, and all Agencies for the Care of the Sick, Criticism and News.

SATURDAY, AUGUST 27TH, 1887.

### Notice to Advertisers.

The scale of charges for small prepaid Advertisements—Situations Wanted, Situations Vacant, etc., etc., is as follows :—

| | s. | d. |
|---|---|---|
| One insertion of three lines | 1 | 0 |
| Three „ „ „ | 2 | 6 |
| Per line afterwards | 0 | 6 |

*A line averages eight words.*

All copy for Advertisements must be sent to A. P. Watt, 2, Paternoster Square, E.C., by first post on the Tuesday *preceding* publication.

### NOTICE TO CORRESPONDENTS.

All correspondence must be written on one side of the paper only.
We cannot undertake to return MSS. not used.
**Communications**, whether intended for insertion or for private information, must be authenticated by the names and addresses of the writers, not necessarily for publication.
**Local papers** containing reports or news paragraphs **should be marked.**
It is especially requested that early intelligence of **local events of interest**, or which it may be thought desirable to bring under notice, may be sent direct to the Editor.
Communications respecting editorial matters should be addressed to the Editor. Norfolk House, Norfolk Street, Strand, London, W.C.

### The Literary Prize.

A PRIZE of **Two Guineas** will be given every month for the best story or incident based upon hospital, or asylum, or student life, or any incident connected with the professions of medicine or nursing.

*** The Children's Corner and Amusements with Profit will be found on page vi of this week's issue.

### THE CHILDREN'S CORNER.

**Fourth Quarterly Prize Competition.**
Began 2nd July, 1887 ; ends 24th Sept., 1887.

**Puzzles—Ninth Week.**

No. 34. ORNITHOLOGICAL ENIGMA.
1. Which is the bird that's seldom seen
By day, yet oft by night ;
2. And he who on the battlefield calls others to the fight ?
3. If you should talk which is the bird
That each word will repeat ?
4. In town or country which is he
Which every day you meet ?

No. 35. DECAPITATION. I am food for cattle ; behead me, and I am the French for storm ; behead me again, and I am in a fury ; behead me again, and I am a period.

No. 36. TRANSPOSITION PUZZLE. Each of the following rows of letters when transposed in proper order, will form a town, and their initials one of the United States :

| | |
|---|---|
| 1. A a b m n o t u n. | 5. D d e l n n o o r r y. |
| 2. A b d e e e n r. | 6. A a a a b l l d h. |
| 3. A a e n n r v. | 7. R r a a n e v. |
| 4. L o v y i e. | 8. A a d d m r s t t. |

No. 37. RIDDLE-ME-REE. What is that which has a mouth and never speaks, and a bed in which it never sleeps ?

**Letters—Ninth Week.**

Next week, let me know your favourite way of spending a wet day in the holidays."

**Results of Seventh Week—Puzzles.**

No. 26. DECAPITATIONS. Prussia, *Russia*, Fowl, *Owl*, Spain, *Pain*, Flint, *Lint*.
No. 27. CHARADE. He, her, hero, heroine.
No. 28. FRENCH PUZZLE. The answer to this is
A long sous P, J grand, a petit.=
Allons souper, j'ai grand appêtit.
No. 29.
Jack and Jill went up a hill
To fetch a pail of water,
Jack fell down, and broke his crown,
And Jill came tumbling after.

*All right*—Umslopogaas, Scrooge ; *Three right*—Toby, Mount Edgcumbe, Agatha, A. C. K. ; *Two right*—Skye, Ossian, The Eda, A. G. S. McCulloch ; *One right*—Fern, Princess Ida, Poodle, Socrates.

**Letters.**

The letters this week give me much pleasure, and I am delighted to hear how all are enjoying their holidays, some at home, some in the country, and others at the seaside. Here is a letter from Skye, describing the delights of Cornwall:—

We are spending our holidays in Cornwall, within twenty minutes' walk of Land's End, and within sight of the Scilly Isles. I am enjoying myself more than I ever did before. We only came on Saturday, and spent the first day and night in Penzance (a beautiful town completely built of granite, and the birthplace of Sir Humphry Davy). We had travelled all day, and only arrived in Penzance at n.ne o'clock, and we could not get *one* cabman to have the hardihood to drive us eight miles into the country on a lonely road, so we had to stay over the Sunday in Penzance. We are living at a nice farmhouse, with kind Cornish people, very near the sea, and surrounded by moors. They look rather bare, but are covered with splendid heather and some very rare ferns, such as the *Royal Fern* and the *Hart's Tongue*, which is as common as grass in some places The nearest post-office is two miles off, and we have to go with and for our letters and papers. The sea-coast is splendid. Great boulders of granite and high cliffs, then patches of beautiful sand, in which are a great many tiny little shells of all colours, but quite perfect. I had a lovely bathe to-day, and tried hard to swim. I did about two yards, and got my mouth full of salt water. I will try again to-morrow. The Cornish people are very kind, and have some very funny customs

Toby sends us an account of Epsom, and the country round :—

I have been spending my holidays partly at Lee and partly at Epsom. While I was at the latter place the annual fair was held. There were steam merry-go-rounds and Aunt Sallies, two or three shows (Pepper's Ghost among them), and a boxing tent. This year, however, it was rather a failure, and instead of lasting for a week, it was all over in three days. The surrounding country is very pretty, and from the top of the Downs a splendid view can be obtained. We went out and picnicked several times. On one occasion we lit a fire to boi. the tea-kettle, and were taken for gipsies by a lady of our acquaintance who passed by. There is also a large medical college, situated on the Downs. During the Derby week the town is filled to overflowing ; the cabs are packed eight deep in the main street, and it is impossible to cross the road without the assistance of a policeman ; but from two till four o'clock in the afternoon, when the principal races are being run, you might walk through all the streets of the town without meeting anybody. Lee seems a pretty little place, but as I have not been very well since I came here, I have not yet seen much of it. We are not very far from the Crystal Palace, where I spent a very pleasant day last week. I have also had one very pleasant walk to Bexley with my uncle. We went through Mottingham, Eltham, and Sidcup, skirting Chislehurst. The country around was lovely, and the hop gardens, of which we passed through one, were beginning to look very pretty indeed.

I hope Ellie will soon have the park she so much wishes for. She writes :—

I am spending my holidays at home. I live close to Brixton Hill, where there is a fine old parish church. We have many places of interest—Raleigh Hall, for instance, said to have been in the possession of Sir Walter Raleigh, but it is now to let, and there is some talk about trying to turn it into a public park, for there are beautiful grounds adjoining. I should be glad if this plan could be carried out, as we are sadly in want of a nice large park to play in.

Three marks each :—Skye, Toby, Socrates, Princess Ida, Ellie. Two marks each :—Ossian, Mount Edgcumbe.

**Notices to Correspondents.**

SOCRATES.—I am very sorry you received THE HOSPITAL so late last week. Thank you for the puzzle.

WALTON-ON-THAMES.—You forget to finish your letter.

Answers must be addressed to the "PUZZLE EDITOR," THE HOSPITAL, Norfolk House, Norfolk Street, Strand, W.C., not later than Thursday next. The "Children's Corner" Coupon (see advertisement pages) must be enclosed.

### AMUSEMENTS WITH PROFIT.

### PRIZE COMPETITIONS.

**Quarterly Prize Competition.**
Began 23rd July ; ends 15th October.

**No. 11. Double Acrostic.**

" On Thames's bank, in silent thought we stood,
Where smiles my *first* upon the silver flood ;
Struck with the seat that gave my *second* birth,
We kneel, and kiss the consecrated earth."

1. Pheasants and hares my first are reckon'd,
2. Sturgeon is deem'd as a dish my second ;
3. The old man's grief was sore ; he fell and died,
4. Another's grief to assuage he vainly tried ;
5. This Fall, the biggest in the world,
6. This swimmer to destruction whirl'd ;
7. Sweet character, in thy disguise,
8. Full many a rogue to do me tries ;
9. Early to bed, and early to rise,
Is the way to my last ; and makes " wealthy and wise."

**No. 12. Sympathy.**

Give, in prose or verse, a definition of *Sympathy*. Answers not to exceed one hundred words

**Answers.**

No. 7. DOUBLE ACROSTIC.

| | | |
|---|---|---|
| H | unneri | C |
| A | murat | H |
| N | | O |
| D | upanlou | P |
| E | nnu | I |
| L | aocoo | N |

Correct answers have been received from Boss, Aralc, Toby, Ostrich, Mater, Brownie, Condorcet, Gretta, Ellice, Man of the Sea.

**No. 8. Enigma.**

We have received *Finger-post* and *Gibbet* as the answer to the above, and, as they apply equally well, we accept both solutions.

Correct Answers have been received from Ellice, Gretta. Condorcet, Brownie, Mater Ostrich, Toby, Aralc, Boss, Esau, V. W., Perseverance, Common Sense, K. M., Fred C., Man of the Sea.

Answers must be addressed to THE PRIZE EDITOR, AMUSEMENTS WITH PROFIT, THE HOSPITAL, Norfolk House, Norfolk Street, Strand, W.C., not later than the *first* post on Friday next. The full name and address must in all cases be given, but a *nom de plume* may be added for publication. Only one side of the paper should be written on. The "Amusements with Profit" Coupon (see advertisement pages) must be enclosed with replies.

